# The BC Cancer Agency Compassionate Access Program: outcome analysis of patients with esophagogastric cancer

**DOI:** 10.3747/co.v16i5.369

**Published:** 2009-09

**Authors:** K.S. Wilson, J.B. Barnett, A. Shah, K.E. Khoo

**Affiliations:** * Department of Medicine, University of British Columbia, Vancouver, BC; † Gastrointestinal Tumour Group, BC Cancer Agency–Vancouver Island Centre, Victoria, BC; ‡ Gastrointestinal Tumour Group, BC Cancer Agency–Vancouver Centre, Vancouver, BC; § Gastrointestinal Tumour Group, BC Cancer Agency–Centre for the Southern Interior, Kelowna, BC

**Keywords:** Esophagogastric adenocarcinoma, chemotherapy, toxicity, survival

## Abstract

**Background:**

The BC Cancer Agency Gastro-intestinal Tumor Group supports one standard of care (soc) chemotherapy regimen for metastatic esophagogastric adenocarcinoma—specifically, weekly cisplatin and 5-fluorouracil (5fu) infusion. All other regimens require Compassionate Access Program (cap) approval for public funding.

**Objectives:**

We examined response, toxicity, and survival after first-line cap chemotherapy (cap1), or soc and second-line cap chemotherapy (cap2).

**Patients and Methods:**

We searched cap records for December 1999 to April 2006, abstracted charts, constructed a database, and undertook survival analyses. Treatment response, serious toxicities, and hospitalizations were recorded.

**Results:**

We identified 32 esophageal (10 gastroesophageal junction) and 53 gastric cancer (62%) patients, 55 of whom were stage M1 at diagnosis. Prior therapy consisted of chemoradiotherapy (*n* = 14), adjuvant chemotherapy (*n* = 3), and radical surgery (*n* = 34). Of these 85 patients, 50 received cap1, and 35 received soc, then cap2. Docetaxel and irinotecan regimens accounted for 34% and 36%, 5% and 55%, 16% and 32% respectively of first-, second-, and third-line cap requests. Partial responses were documented with soc (11/35, 31%) and cap1 (6/50, 12%). Grade 3+ toxicity rates were 19/50 (38%) and 6/35 (17%) with cap1 and soc chemotherapy. With cap chemotherapy, 20 hospitalizations occurred, and with soc chemotherapy, 2 hospitalizations. For all patients, median follow-up and survival times were 8.9 months and 9.7 months respectively.

**Limitations:**

This is a retrospective analysis of patients deemed suitable to receive non-soc chemotherapy regimens or unsuitable to receive soc chemotherapy.

**Conclusions:**

Toxicities in cap chemotherapy regimens were substantial. Survival times were consistent with results of international phase ii and iii trials in esophagogastric cancer.

## 1. INTRODUCTION

Implementation of new chemotherapy protocols at the BC Cancer Agency (bcca) is subject to approval by the Priorities and Evaluation Committee (pec) and the Systemic Therapy Program (stp). Reports of the effectiveness of new therapies in journals and conference proceedings lead to their evaluation by bcca tumour groups, whose members advance appropriate protocols to pec for approval and to stp for funding. Normally, phase iii clinical trial data with adequate follow-up—or meta-analyses—are required to support major new programs. For less common malignancies, only phase ii data may be available. In the period between the results of phase ii and iii trials, or before pec and stp approval, utilization of evolving therapies may be requested through the Compassionate Access Program (cap).

Esophagogastric adenocarcinoma treatment has progressed in the past decade to include adjuvant and primary chemoradiotherapy for esophageal cancer 1,2 and postoperative chemoradiotherapy for gastroesophageal junction and gastric adenocarcinomas 3. These results led to the introduction at bcca of giefuprt [cisplatin, infusional 5-fluorouracil (5fu), radiation therapy] and gigairt (5-fluorouracil, folinic acid, and radiation therapy) regimens for esophageal and gastric cancers respectively 4,5 (Table I). For incurable metastatic esophagogastric adenocarcinoma, the bcca Gastro-intestinal Tumour Group supports one standard-of-care (soc) chemotherapy regimen incorporating weekly infusional 5fu and cisplatin (gifuc 6,7). All other regimens require cap approval for public funding. The present report describes the bcca experience with various regimens for metastatic esophagogastric adenocarcinoma in the second line after soc chemotherapy (cap2), or in the first line instead of soc (cap1). The principal objective of this type of practice review is to assist future treatment decision-making by assessing the total provincial patient experience, which would otherwise be unknown to individual physicians.

## 2. PATIENTS AND METHODS

This retrospective review was approved by the Research Ethics Board of the bcca and University of British Columbia. It includes patients with surgically incurable esophagogastric adenocarcinoma who received a chemotherapy regimen through the cap either as initial therapy (cap1) or in the second line (cap2) after the current soc regimen of cisplatin and infusional 5fu 6,7. Patients were identified from the database of the bcca cap, which approves funding and collects data prospectively on these patients. The bcca clinical records and local hospital records were retrieved, and the relevant clinical and pathology information was abstracted into a separate database for analysis. The time period was 77 months from December 1, 1999, through April 30, 2006.

Database fields included date of birth and sex; disease stage at diagnosis, primary site, and date of diagnosis; prior surgical, radiotherapy, and adjuvant chemotherapy status; initial and subsequent chemotherapy regimen or regimens; start and finish dates for chemotherapy; worst toxicity grade and type recorded; number of hospital days attributable to chemotherapy complications; response category if documented; date of last follow-up and patient status at that time. Overall survival was defined as time from diagnosis until death from any cause, calculated using the Kaplan–Meier method 8. Data were analyzed using the Statistical Software Package for the Social Sciences (SPSS, version 10.1 for Windows: SPSS, Chicago, IL, U.S.A.).

## 3. RESULTS

A total of 85 patients [61 men (72%), 24 women; 68 Caucasian, 15 Asian, 2 Fijian; median age: 56.2 years (range: 28.7–81.8 years)] received cap chemotherapy regimens for esophagogastric adenocarcinoma (Table II). Disease stage was M1 in 55 patients at diagnosis. The primary tumours were gastric (*n* = 53) and esophageal (*n* = 32), with 10 gastroesophageal junction tumours in the latter group. Prior therapies included radical surgery (*n* = 34), chemoradiotherapy (*n* = 14), and adjuvant chemotherapy (*n* = 3). Of the patients who received cap1 chemotherapy (*n* = 50), 25, 9, and 2 received second-, third-, and fourth-line chemotherapy respectively. Reasons for using cap1 included failure of prior adjuvant therapy [*n* = 13: folfiri (5fu, irinotecan, leucovorin), 5; dcf (docetaxel, cisplatin, 5fu), 3; other regimens, 5], physician preference [*n* = 22: dcf, 8; folfiri, 8; giefup (cisplatin, infusional 5fu, with or without radiation therapy), 6, of whom 2 received concomitant radiation therapy], and patients unsuitable for, or declining, cisplatin or infusional 5fu (*n* = 15). There was a trend toward younger age patients receiving cap1 chemotherapy (median age: 55.3 years vs. 60.3 years). In 35 patients, soc was followed by cap2 chemotherapy. Docetaxel and irinotecan regimens accounted for 34% and 36%, 5% and 55%, and 16% and 32% of first-, second-, and third-line cap chemotherapy regimens respectively.

The proportions of patients experiencing grade 3 or 4 toxicity with soc chemotherapy and with cap1 chemotherapy were 5/35 (17%) and 19/50 (39%) respectively. There were 20 hospitalizations attributable to cap1 or cap2, and 2 attributable to soc chemotherapy. Toxicity caused 1 death (folfiri regimen). Partial responses were seen with soc (11/35) and cap1 (6/50) chemotherapy. Second-line chemotherapy produced 4 responses: 2 after failing soc, and 2 after failing cap1. Median follow-up was 8.9 months.

**FIGURE 1:**
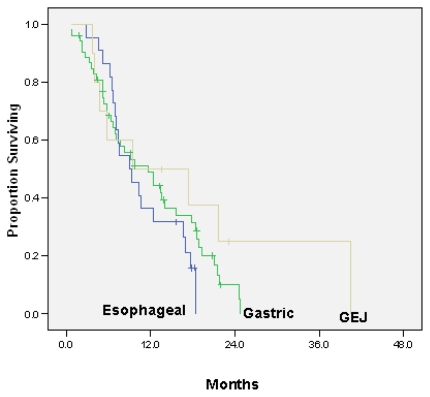
Kaplan–Meier analysis of overall survival by primary tumour site.

**FIGURE 2:**
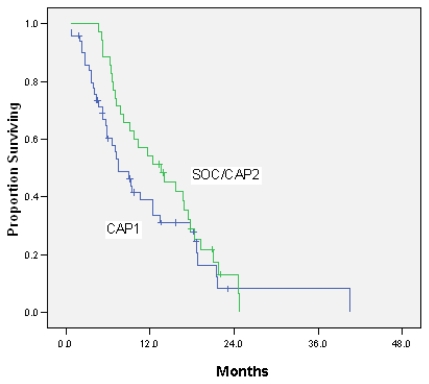
Kaplan–Meier analysis of overall survival (os), comparing cap1 with standard of care (soc ) and cap2 chemotherapy. Median os was 7.5 months (cap1) and 13.6 months (soc and cap2).

Median survival time for all patients was 9.7 months [95% confidence interval (ci): 6.2 to 13.1 months]. Figures 1 and 2 present survival analyses according to primary site and cap1 or cap2 chemotherapy. Median survival for patients who had received no prior chemotherapy (*n* = 68) was 11.6 months (95% ci: 8.4 to 14.9 months); for those who had received prior chemotherapy, it was 5.2 months (95% ci: 2.4 to 7.9 months). Median survival for patients over 50 years of age at diagnosis (11.6 months) was longer than that for patients under 50 years of age (6.6 months).

## 4. DISCUSSION

New chemotherapy regimens have been extensively studied in esophageal and gastric adenocarcinomas 9–14. Improvements in survival have been demonstrated in phase iii trials in gastric cancer, but only phase ii trials have been undertaken in metastatic esophageal cancer, mainly because of small numbers of suitable patients. Patients with gastroesophageal junction cancer are sometimes included in gastric cancer trials on the assumption that those diseases are biologically similar. With the increasing use of adjuvant regimens in esophagogastric cancer, more patients with advanced disease have been previously exposed to at least a fluoropyrimidine.

Debate continues in North America about the relative merits of newer regimens in metastatic esophagogastric cancer, especially when survival differences are small and toxicity rates are equal or greater 15,16. For example, as compared with cf (cisplatin, 5fu) in advanced gastric cancer, dcf yields a longer median survival (9.2 months vs. 8.6 months) and a better 2-year survival rate (18% vs. 9%), which led to its approval by the U.S. Food and Drug Administration for gastric and gastroesophageal junction cancers 9. Grades 3 and 4 non-hematologic and neutropenic toxicity rates were 81% and 84% respectively with dcf 9. The editorial that accompanied publication (“Does the punishment fit the crime?”) reflects the balance required between survival gains and toxicities experienced 17. Overall survival after ecf (epirubicin, cisplatin, 5fu) chemotherapy was longer than that with famtx (doxorubicin, 5fu, leucovorin, methotrexate: 8.9 months vs. 5.7 months) at the cost of more nausea, vomiting, and alopecia, and fewer episodes of neutropenia and infection 10. Survival after folfiri was equivalent to that after cf, but more grade 3+ diarrhea occurred with folfiri (22% vs. 7%) and more grade 3 neutropenia occurred with cf (52% vs. 25%) 12. There is no standard second-line chemotherapy regimen for gastric cancer after failure of either adjuvant or first-line chemotherapy 16.

In metastatic esophageal cancer, chemotherapy is usually limited to patients with a good performance status (Eastern Cooperative Oncology Group ≤ 2), preferably in a clinical trial. A regimen of cisplatin–5fu has been used most frequently. Other regimens include ecf, irinotecan–cisplatin, paclitaxel–cf, paclitaxel–carboplatin, gemcitabine–cisplatin, folfiri, and capecitabine (in place of 5fu)–oxaliplatin. Two regimens are the recommended maximum, because of a lack of a proven survival benefit. Regimen choice depends in part on prior chemotherapy received, organ function status (especially renal status), and issues of venous access 15.

Integration of new or nonstandard chemotherapy regimens into clinical practice occurs in response to evolving clinical trial results, adequate levels of evidence, and availability of drugs with differing modes of action and toxicity profiles. Typically, the institutional process for approval of a new regimen or indication takes several months, during which time cap access may be available. Physicians may apply to use a novel regimen in the first line if there are any clinical contraindications to the current bcca soc. However, many regimens shown to be active in phase ii trials are not advanced to full systemic therapy approval because of infrequent usage.

Our experience with the use of nonstandard regimens in the first line (cap1) in metastatic esophagogastric cancer is reported in conjunction with that in patients on soc who later receive cap regimens (cap2). Both groups of patients are inevitably “selected” in terms of reasons for cap1 use—for example, better performance status in some cases (dcf regimen), diminished organ function (for example, favouring irinotecan regimens over cisplatin in the presence of renal dysfunction), or failure of prior adjuvant therapy or radical chemoradiotherapy. Furthermore, patients were all deemed sufficiently fit to receive both cap1 and cap2 regimens. Notably, only 50% of cap1 patients received secondary therapies; by definition, all cap2 patients had received soc therapy first. Hence there is potential bias in both directions.

Response assessments in retrospective analyses such as the present one are problematic, because they are not protocol-mandated and are subjective in terms of symptom and palliative benefit. Unlike the 20% seen in the present series, few or no patients had received prior chemotherapy in most published trials (Table III). The hard endpoint is survival, and the observed survival time (median: 9.7 months) and proportion (10.4% at 2 years) in the present series are consistent with contemporary clinical trials. Median survival after gifuc chemotherapy in 205 patients across British Columbia between 2001 and 2006, 25% of whom received second-line or subsequent chemotherapy, was 11.2 months 18. Conservatively interpreted, this survival appears to be at least as good as the median survival for all patients in the present series (9.7 months) and for those who received cap1 chemotherapy (7.5 months).

An examination of toxicity rates between soc and cap1 patients is subject to less potential bias. Despite patients being selected as suitable for cap therapies, severe toxicity and hospitalization rates were higher with cap1 therapy. Although the negative resource and quality-of-life impacts of these events are self-explanatory, they may have affected survival either positively or negatively. Severity of side effects of chemotherapy has been associated with higher response rates and longer survival in advanced colorectal cancer 19. However, until this association is proven prospectively in esophagogastric cancer, the merits of first-line therapy with other than fluorouracil–cisplatin should be carefully considered in view of the toxicity encountered.

## 5. CONCLUSIONS

The use of nonstandard chemotherapy regimens for surgically incurable esophagogastric adenocarcinoma in British Columbia has been examined and outcomes have been analysed. To put their own experiences into perspective, individual physicians need population outcome analyses of the kind that this review provides. Caution is, of course, required in interpreting retrospective nonrandomized patient cohorts. Nevertheless, nonstandard chemotherapy regimens have substantial toxicities that should be discussed with patients as part of the decision process in their use.

## 7. CONFLICT OF INTEREST

No external funding was obtained for the purpose of this review. The authors declare no conflicts of interest. This review was published in abstract form at the American Society of Clinical Oncology Annual Meeting 2006.

## Figures and Tables

**TABLE I:** BC Cancer Agency esophagogastric cancer protocols

Protocol	5-Fluorouracil	Cisplatin	Folinic acid	Cycle
gifuc	1000 mg/m^2^ infusion, daily for 2 days	25 mg/m^2^ once, day 1	—	7 days
giefuprt	1000 mg/m^2^ infusion, daily for 4 days	25 mg/m^2^ for 3 days	—	28 days
gigairt	425 mg/m^2^ bolus, daily for 5 days		20 mg/m^2^ daily for 5 days	28 days

**TABLE II:** Patient characteristics by Compassionate access Program (cap) usage

	Pts (*n*)	Men [*n* (%)]	Median age (years)	Prior chemotherapy [*n* (%)]	M1 at diagnosis [*n* (%)]
cap1	50	38 (76)	55.3	16 (32)	29 (58)
soc/cap2	35	23 (66)	60.4	1 (3)	27 (77)

Pts = patients; cap1 = cap used in the first line; soc = standard of care; cap2 = cap used in the second line.

**TABLE III:** Clinical trial results of chemotherapy in metastatic esophagogastric cancer

Reference	Regimen	Site	Pts (n)	Prior chemotherapy	Median survival (months)	95% ci	2-Year survival (%)	Quality of life	trm (%)	Adenocarcinoma (%)
Webb *et al.,* 1997^10^	ecf	Esophagogastric	111	No	8.9	nr	11	ecf>famtx	0.9	100
	famtx		108	No	5.7	nr	6		1.9	
Ross *et al.,* 2002^11^	ecf	Esophagogastric	289	No	9.4	nr	15.8	ecf>mcf	0.3	93.1
	mcf		285	No	8.7	nr	14.2		0.4	93.0
Pozzo *et al.,* 2004^14^	folfiri	Gastric	59	5.4%	10.7	8 to 14.6	13	folfiri>iri+c	0	100
Dank *et al.,* 2005^12^	folfiri	Esophagogastric	170	Permitted	9	8.3 to 10.2	14	Trend favouring folfiri	0.6	100
	cf		163		8.7	7.8 to 9.8	12		3	
Roth *et al.,* 2005^13^	dcf	Gastric	41	No	10.4	8.3 to 12	nr	ecf>dcf	0	100
	ecf		40	No	8.3	7.2 to 13	nr		0	
Van Cutsem *et al.,* 2006^9^	dcf	Gastric	221	No	9.2	8.4 to 10.6	18	dcf>cf	2.7	100
	cf		224		8.6	7.2 to 9.5	9		4.5	
BC Cancer Agency, this report		Esophagogastric	85	20.00%	9.7	6.2 to 13.1	10.4	nr	1.2	100

Pts = patients; ci = confidence interval; trm = treatment-related mortality; ecf = epirubicin, cisplatin, 5-fluorouracil; famtx = doxorubicin, 5-fluorouracil, leucovorin, methotrexate; nr = not reported; mcf = mitomycin, cisplatin, 5-fluorouracil; folfiri = 5-fluorouracil, irinotecan, leucovorin; iri+c= irinotecan + cisplatin; cf= cisplatin, 5-fluorouracil; dcf= docetaxel, cisplatin, 5-fluorouracil.
